# A comparison of clinical profile and treatment outcome of Hodgkin’s Lymphoma in Tanzania according to HIV status during the HAART era

**DOI:** 10.1186/s12885-024-12569-z

**Published:** 2024-07-03

**Authors:** Mercy M. Mbai, Emmanuel Mduma, Samuel Thuo, Eulade Rugengamanzi, Christina V. Malichewe, Emmanuel L. Lugina

**Affiliations:** 1 Department of Clinical Oncology, Muhimbili University of Health and Allied Sciences, Dar Es Salaam, Tanzania; 2 Department of Oncology, Longisa County Referral Hospital, Bomet, Kenya; 3 Department of Clinical Oncology, Rabininsia Memorial Hospital, Dar Es Salaam, Tanzania; 4 Department of Otolaryngology, Muhimbili University of Health and Allied Sciences, Dar Es Salaam, Tanzania; 5 Butaro Cancer Center of Excellence, Burera, Rwanda; 6 School of Medicine, University of Global Health Equity, Kigali, Rwanda; 7 Academic, Research and Consultancy Unit, Ocean Road Cancer Institute, Dar Es Salaam, Tanzania

**Keywords:** Hodgkin’s Lymphoma, Clinical profile, Overall survival, Predictors of survival, HIV

## Abstract

**Background:**

The incidence of Hodgkin's lymphoma (HL) in people living with HIV (PLWHA) and on HAART is approximately 20–30 times higher than in HIV-negative individuals. Most patients with HIV-HL present at an advanced stage (III-IV) have 'B' symptoms and extranodal involvement. The natural history and risk stratification of HIV-HL has undergone a significant change as a result of HAART's rollout. This study investigated the differences in clinicopathological and survival patterns of HL among individuals with and without HIV disease in Tanzania during the HAART era.

**Methodology:**

This hospital-based retrospective cohort study was conducted at the ORCI, Dar-Es-Salaam, Tanzania. Chi-square and Fisher’s exact tests were used to compare proportions. The student t-test was used to compare means. To determine factors that predict survival, we used the log-rank test to analyze the variables in univariate analysis. A Cox regression model was used to analyze the significant factors from univariate analysis in multivariate analysis.

**Results:**

Eighty-three patients with HL were recruited, and the prevalence of HIV-positive status was 27.7%. Most of the patients with HIV-HL had an age of > 30 years (73.9%), while most of the non-HIV-HL patients had an age of ≤ 30 years (63.3%) (*P* = 0.02). The 2-year OS rate for HIV-HL was 34%, while that for non-HIV-HL was 67%. Among the HIV-HL patients, predictors of a poorer outcome were a CD4 count ≤ 200 cells/mm3 (*P* = 0.05), lack of HAART use (*P* = 0.00), and the use of HAART for ≤ 10 months (*P* = 0.00).

**Conclusion:**

The prevalence of HIV-HL was 27.7% among HL patients. HIV positivity is still a poor prognostic factor in our setting, especially for patients not on HAART, on HAART for ≤ 10 months, or with a low CD4 count below 200 cells/mm3. Patients with HIV-HL were older and had higher LDH levels, whereas patients with non-HIV-HL were younger and had low LDH levels.

## Introduction

Hodgkin lymphoma (HL; formerly called Hodgkin's disease) is a germinal center, B-cell malignant disorder that affects the reticuloendothelial and lymphatic systems [1].

Worldwide, according to Globocan 2020, HL was ranked 26th in new cases with 83,087 new cases and 27th in causes of death with 23,376 deaths in 2020 [2]. In Tanzania, HL was ranked 22nd in new cases, having 339 new cases, and 24th in the causes of death, with 150 deaths [3].

Human Immunodeficiency Virus (HIV) infection has been associated with an increased risk of lymphomas, especially high-grade B-non-Hodgkin lymphomas (NHL) and Hodgkin’s lymphomas [4]. However, HL is not considered to be an AIDS-defining malignancy.People living with HIV/AIDS (PLWHA) are 3–10 times more likely to develop HL than HIV-uninfected populations. For those receiving HAART, this risk swells to 20–30 times [5]. Previous studies have shown that HAART can restore immune function in PLWH, and the tumor (Reed-Sternberg cells) microenvironment can be supported by increased leukocyte counts in ways that are less likely without HAART [6]. In the US, Biggar et al. found a 9.4 times higher incidence of HL among people with AIDS than in the general population [7].

HIV raises the incidence of HL by inducing progressive immunological suppression [acquired immunodeficiency syndrome (AIDS)] and likely loss of Epstein–Barr virus immunologic control [8].

HIV-HL had a poor prognosis before the HAART era, with only a few patients successfully cured.The negative prognostic factors were systemic "B" symptoms, mixed cellularity subtype, extranodal involvement, and a high International Prognostic Score (IPS). HIV-HL's risk stratification and natural history have been significantly affected by the development of HAART [8].

Recent studies undertaken in the HAART era in high-income countries (HICs), including Britain [9] and Germany [10], have indicated that patients with HIV-HL survival rates are now nearing those reported in HIV-uninfected patients. In Germany, Hentrich et al. found the 2-year overall survival rate of the 108 patients (all HIV positive) included in the study was 90.7% [10]. In Britain, Montoto et al., on the other hand, found that the 5-year OS for non-HIV-HL patients was 88% with no significant differences compared with patients with HIV-HL patients (81%; *P* = 0.15) [9]. In France, from the cohort of 159 patients with HIV-HL, the two-year overall survival was 94% [95%CI 89%,- 100%] [4]. However, in the US, Olszewski et al. still found a significantly lower 5-year unadjusted OS for HIV-HL compared to non-HIV-HL (66% vs 80% respectively) [11].

There being a scarcity of data on HL among PLWHA in low-income countries, this study aimed to determine differences in clinical pathological patterns and survival patterns of HL among people with and without HIV disease in Tanzania in the HAART era.

## Materials and methods

### Study design and subjects

This retrospective cohort study enrolled all consecutive newly diagnosed patients who were histologically confirmed HL aged 18 years or older and both sexes diagnosed and treated at the Ocean Road Cancer Institute (ORCI), Dar-es-Salaam, Tanzania, from Jan 2016 to Dec 2019. All patients with second malignancy, unknown HIV status, a lot of missing data, or those who used another chemotherapy regimen apart from ABVD were excluded from this study. Baseline and follow-up data were extracted from the patient’s medical records using a data extraction tool. There were no patients whose HIV status changed in the course of treatment/during follow-up, and all were analyzed according to their baseline HIV status. HL patients who met inclusion criteria were followed for two years, and the endpoint was 2 years of overall survival (OS).

Ethical clearance for conducting this study was sought from the Muhimbili University of Health and Allied Sciences (MUHAS) Ethical Review Board (approval number: MUHAS-REC-12–2021-907). The approval to carry out the study was sought from the ORCI Ethical Review Board. Considering the study's retrospective nature, informed consent was waived from the MUHAS ethical review board.

### Treatment protocol

The treatment protocol for HL at the ORCI incorporates both chemotherapy and involved field radiotherapy. The first-line chemotherapy used was ABVD for sixcycles(Adriamycin 25 mg/m2, Bleomycin 10 mg/m2, Vinblastine 6 mg/m2, Dacarbazine 375 mg/m2).Involved field radiotherapy is given at 1.8 Gy/fraction for 17–20 fractions. Radiological investigations, including CT scans of the neck, chest, abdomen, and pelvis, were mainly done for staging during diagnosis, assessment of treatment response, and follow-up. ORCI was not equipped with a PET-CT scan. Post-treatment follow-up at ORCI is three monthly in the first two years, then six monthly for three years, and annually afterward.Platinum-based chemotherapy was given to the majority of patients who relapse.

### Statistical analysis

Statistical Package for Social Science (SPSS v.25) was used for statistical analysis. The Kaplan–Meier method was used to draw survival curves.Quantitative variables were summarized using means and standard deviations, while qualitative variables were summarized using proportions.Comparing proportions was done using Chi-square and Fisher's exact tests.A student t-test was used to compare means.The log-rank test was used for univariate analysis to identify factors that predict survival. Factors with a *P*-value of less than 0.2in univariate analysis were assessed by multivariate analysis using a Cox regression model.Missing data was excluded from the analysis. Overall survival (OS) was defined as the time from diagnosis until death or the end of follow-up. Patients who were lost to follow-up and patients who were alive at the end of the study period were censored. A *P*-value of less than 0.05 was considered statistically significant.

## Results

A total of 121 subjects with a diagnosis of HL were retrieved; however, the study enrolledeighty-three patients who met the eligibility criteria.The prevalence of HIV-positive status was 27.7%.

**Figure:**
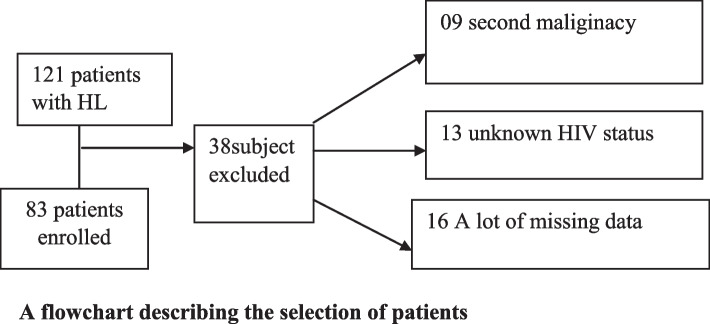
A flowchart describing the selection of patients

The female-to-male ratio was 1:1.8. The mean age of HIV-HL patients was 38.7 ± 10.8 years, and that of non-HIV-HL was 31.6 ± 15.6 years, but the difference was not statistically significant using the t-test. The maximum age was 79 years, while the minimum age was 18.

The mean CD4 cell count among HIV-HL patients was 242 cells/mm3. The mean hemoglobin level among HIV-HL patients was 9.5 ± 2.6 g/dL, and that of non-HIV-HL patients was 10.2 ± 2.8 g/dL, but the difference was not statistically significant using the t-test.

Most HIV-HL patients (76.2%) had LDH levels of > 500 U/L, and most non-HIV-HL (58.8%) had LDH levels of ≤ 500 IU/L, and the difference was statistically significant. The difference between HIV-HL and non-HIV-HL in their mean LDH levels was statistically significant (*P* = 0.01), with HIV-HL having a mean LDH level of 691 IU/L and non-HIV-HL having a mean LDH level of 491 IU/L. The upper limit of LDH levels at ORCI is 325 IU/L (Table 1).

**Table 1:** Patient characteristics (*N* = 83)

	**HIV-HL** **Number *n* = 23(%)**	**Non-HIV-HL** **Number *n* = 60(%)**	***P*-Value**
Gender			
Male (*n* = 53)	13 (56.50)	40 (66.7)	0.2
Female (*n* = 30)	10 (43.5)	20 (33.3)	
Age (Years)			
≤ 30 (*n* = 44)	6 (26.1)	38 (63.3)	0.02
> 30 (*n* = 39)	17 (73.9)	22 (36.7)	
Health insurance status			
Yes (*n* = 24)	5 (20.8)	19 (31.7)	0.3
No (*n* = 59)	18 (78.2)	41 (68.3)	
CD4 Count (missing data = 70)			
≤ 200 (*n* = 5)	5 (38.5)		
> 200 (*n* = 8)	8 (61.5)		
Histology			
Nodular sclerosis (*n* = 22)	10 (43.5)	12(20.2)	0.1
Mixed cellularity (*n* = 30)	8 (34.8)	22 (36.7)	
Lymphocyte rich (*n* = 7)	2 (8.7)	5 (8.3)	
Lymphocyte depleted (*n* = 7)	2 (8.7)	5 (8.2)	
Unknown (*n* = 13)	0 (0)	13 (21.7)	
Nodular lymphocyte-predominant (*n* = 4)	1 (4.3)	3 (5.0)	
Primary site			
Neck Nodes (*n* = 49)	0 (0)	49 (81.7)	0.1
Mediastinum (*n* = 9)	7 (30.4)	2 (3.3)	
Abdomen (*n* = 3)	0 (0)	3 (5.0)	
Disseminated (*n* = 6)	0 (0)	6 (10.0)	
Presence of B Symptoms			
Yes (*n* = 62)	17 (73.9)	45 (75.0)	0.8
No (*n* = 21)	6 (26.1)	15 (25.0)	
Stages			
1&2 (*n* = 43)	10 (43.5)	33 (55.0)	0.3
3&4 (*n* = 40)	13 (56.5)	27 (45.0)	
Hemoglobin			
≤ 9 (*n* = 32)	11 (45.8)	21 (35)	0.3
> 9 (*n* = 51)	12 (54.2)	39 (65)	
LDH Level (IU/L) (Missing data = 11)			
≤ 500 (*n* = 35)	5 (23.8)	30 (58.8)	0.007
> 500 (*n* = 37)	16 (76.2)	21 (41.2)	
ECOG			
1(*n* = 47)	10(43.5)	37 (61.7)	0.3
2(*n* = 26)	9 (39.1)	17 (28.3)	
3(*n* = 10)	4 (17.4)	6 (10.0)	
CD20 (Missing data = 69)			
Positive (*n* = 4)	0 (0)	4 (6.8)	0.5
Negative (*n* = 10)	3 (12.5)	7 (11.9)	
Unknown (*n* = 69)	21 (87.5)	48 (81.4)	

*N* = total sample size and *n* = sample in the subgroup

Most of the patients with HIV-HL (60.9%) were treated with ≤ 4 cycles of ABVD regimen, whereas most of the patients who had non-HIV-HL (70%) were treated with > 4 cycles of ABVD regimen (*P* = 0.01) and the median number of cycles was 6 for the entire cohort.The majority of PLWHA (78%) were on HAART when they were diagnosed with HL. The mean duration of HAART use was 26 months, with a range of 2–120 months (Table 2).

**Table 2:** Treatment characteristics (*N* = 83)

	**HIV-HL** **Number *n* = 23(%)**	**Non-HIV HL** **Number** ***n* = 60(%)**	***P*-Value**
Number of cycles			
< 4	14 (60.9)	18 (30.0)	0.01
> 4	9 (39.1)	42 (70.0)	
IFRT			
Yes	1 (4.3)	11 (18.3)	0.1
No	22 (95.7)	49 (81.7)	
HAART use (*N* = 23)			
Yes	18 (78.0)		
No	5 (22.0)		

**Fig. 1:**
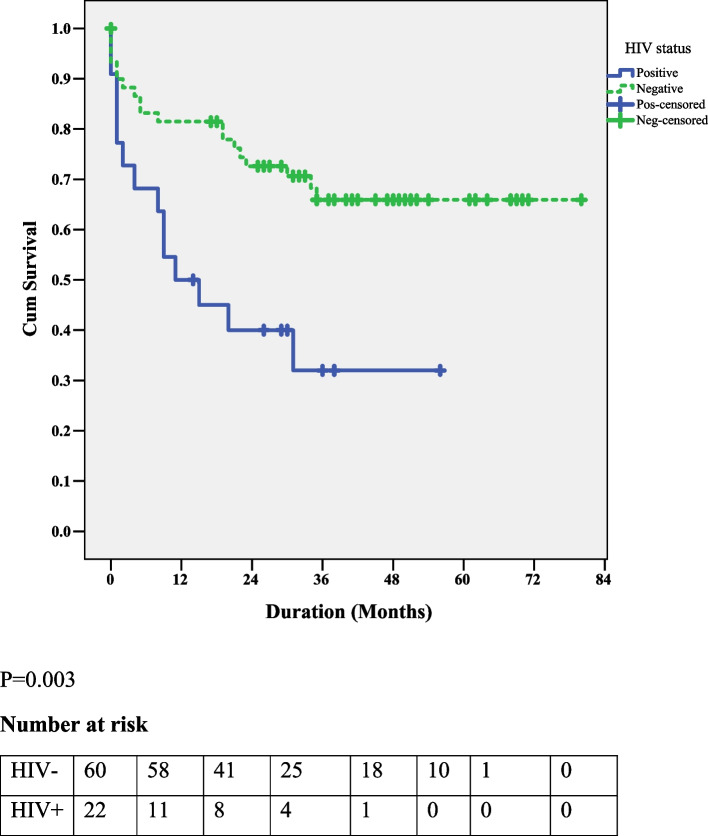
The comparison of OS between HIV-HL and non-HIV = HL

The OS was significantly higher in non-HIV-HL patients compared to HIV-HL patients (*P* = 0.003). The 2-year OS rate for HIV-HL was 34%, while that for non-HIV-HL was 67% (Fig. 1).

**Fig. 2:**
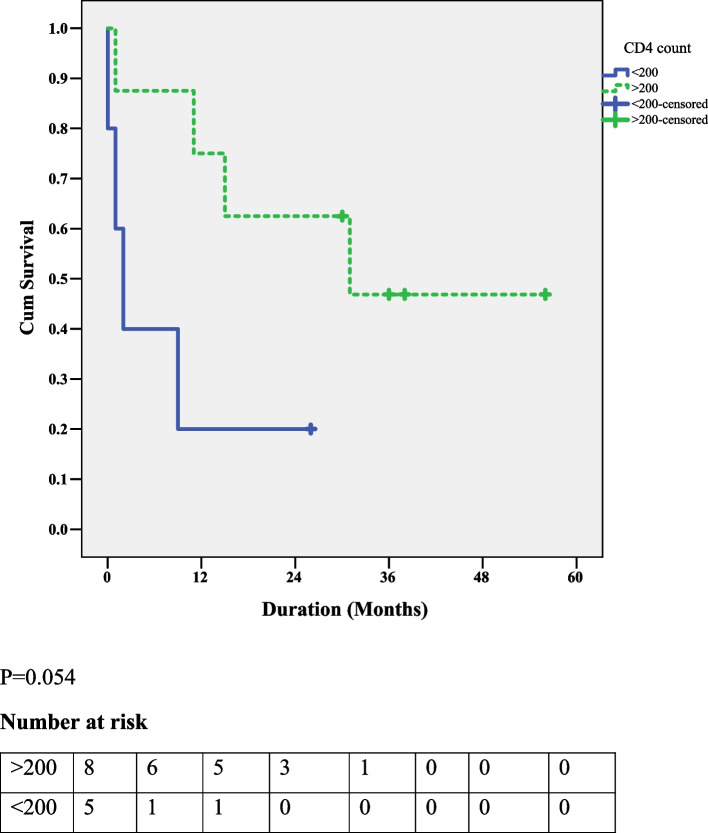
The association between OS and CD4 count (cells/mm3)

Patients with HIV-HL and CD4 count of > 200/mm3 hada higher OS compared to those with a CD4 count of ≤ 200/mm3, and the difference was statisticallysignificant(*P* = 0.054) (Fig. 2).

**Fig. 3:**
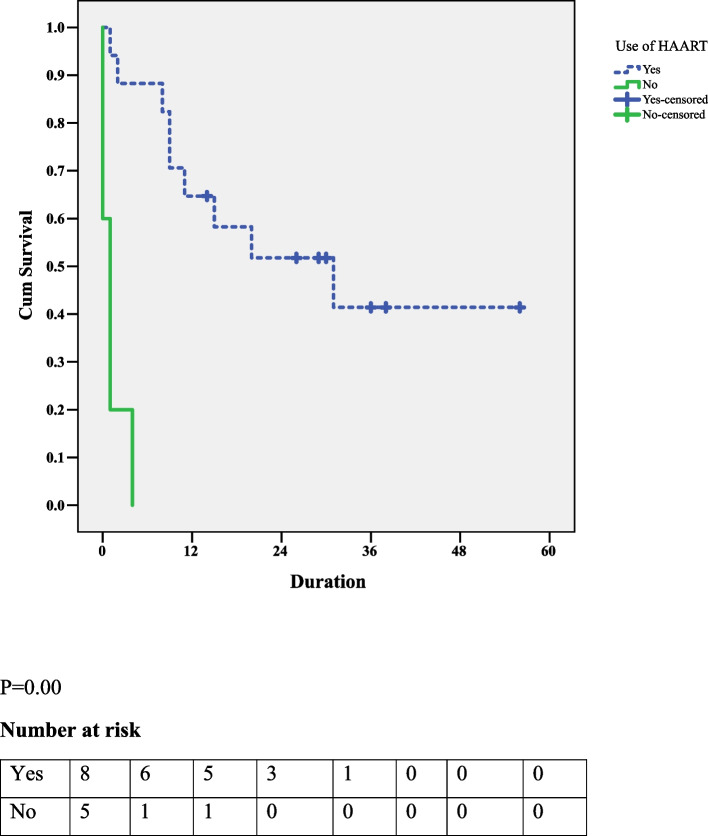
The association between the use of HAART and OS among patients with HIV-HL

Patients with HIV-HL and on HAART have a higher OS than those not on HAART (*P* = 0.000) (Fig. 3).

**Fig. 4:**
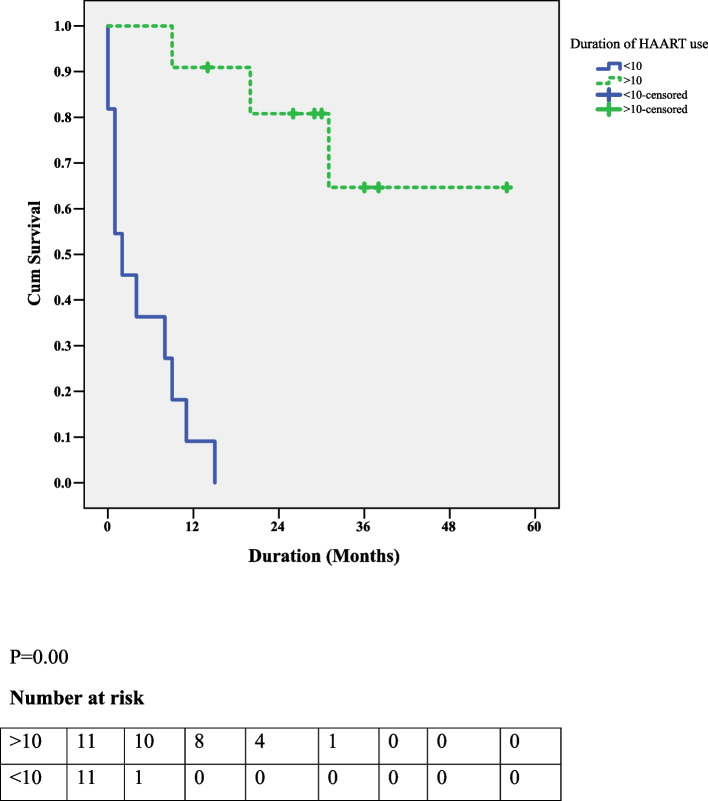
The association between HAART use duration and OS among HIV-HL patients

The median duration of HAART use was ten months.The OSof patients with HIV-HL and on HAART for > 10 months was 31 higher than for those on HAART for ≤ 10 months (*P* = 0.000) (Fig. 4).

The Cox regression analysis showed that LDH level, HIV status, and HL stage were significantly linked to the OS. HL patients with LDH levels of > 500 U/L were 2.5 times more likely to die than those with LDH levels of ≤ 500 U/L (*P* = 0.04). Non-HIV-HL patients had 60% less risk of dying (*P* = 0.023) than HIV-HL patients. HL patients with late stages (3 and 4) were eight times more likely to die in comparison to those with early stages (*P* = 0.000) (Table 3).

**Table 3:** A summary of univariate and multivariate analysis

*Variable*	*P-Value*	*HR*	*95% CI*	*P-value*	*HR*	*95% CI*
***HIV status***	0.00	2.7	1.4–5.5	0.02	2.5	1.1–5.4
***Age groups***	0.42	1.33	0.6–2.6			
***Insurance status***	0.43	0.43	0.6–3			
***LDH Groups***	0.001	0.2	0.1–0.6	0.04	0.4	0.2–0.9
***Early vs. Late stage***	0.001	0.2	0.1–0.4	0.00	0.1	0.04–0.3

## Discussion

The prevalence of HIV-HL was 27.7%in the index study.In high-income countries (HIC), there is a variation in the prevalence of HIV among HL patients.The prevalence was very low in the US (3.8–5%) [8], while in the UK, it was quitehigh (41.5%) from 1997 to 2010 [9]. However, in Africa, studies conducted in South Africa and Malawi found prevalence rates of 25% and 33% respectively [12, 13]. The results in these African countries closely resemble our finding of a prevalence of 27.7%.In Tanzania, the prevalence has decreased significantly over the last 20 years, dropping from 44.4% [14]. This could be attributed to the increased implementation of HIV prevention measures throughout the years, leading to an overall reduction in HIV cases.

The majority of HIV-HL patients (73.9%) were over 30 years old, while most non-HIV-HL patients were under 30 years old (*P* = 0.02).This is consistent with the observation that HIV-HL occurs in older generations in most HICs compared to their non-HIV-HL counterparts [8, 11]. This is likely due to the growing aging population of persons living with HIV since the initiation of HAART, with the risk of acquiring HL in this population still being more than that of the general population [6].

Among patients with HIV-HL, NS was the most common histologic subtype (43.5%), trailed by MC (34.8%). Meanwhile, in non-HIV-HL, MC was the most common histology (36.7%).The index study showed a non-significant difference in histology based on HIV status (*P* = 0.1).Contrary to our findings, in the US and Europe [4, 6, 8, 15], a predominance of MC was found among HIV-HL patients. The index study could have shown a difference in histology distribution between the two cohorts if it had a larger sample size.

The majority (61.5%) of HIV-HL patients with recorded CD4 count showed a CD4 count > 200 cells/mm3 when diagnosed with HL; the average CD4 count was 242 cells/mm3.It has been demonstrated by various studies that HL is more prevalent in patients with moderate levels of immunodeficiency than those with severe immunodeficiency (< 50 cells/mm3) [7, 16]. Carbone et al. found a mean CD4 count of 210 cells/mm3 [17], while Naidoo et al. in South Africa found that 54.6% of the HIV-positive cases had CD4 counts > 150cells/mm3 [12]. Most of the patients were on HAART (78%) at the diagnosis of HL. The use of HAART has been associated with a higher risk of HL; Spina et al. deduced that as the HAART increases the CD4 count levels, it paradoxically raises the occurrence of HL [16]. This deduction accounts for the increase in HIV-HL patients on HAART in this study. An influx of inflammatory and CD4 cells is thought to provide proliferative signals to the neoplastic RS cells [16]. The recruited inflammatory cells may provide essential feedback signals that stimulate proliferation or inhibit apoptosis of RS cells. In severe immunosuppression, it is suggested that HL remains occult until the immune system is sufficiently reconstituted to respond to RS cells [7].

HL is a highly curable disease with a 2-years OS rate approaching 100% in high-income countries(HIC) such as Spain, Germany, and Austria [10, 18]. The 2 years OS rate for the entire cohort in this study was 58%. This aligns with the study in Malawi, where the two-year OS rate was 61% [13]. Adverse baseline characteristics and a severely resource-constrained environment may have contributed to the overall poor treatment outcome. Lack of PET-CT scan at diagnosis may have resulted in under-staging of patients who have advanced disease and inability to identify patients who have refractory disease who needed to be treated with six cycles of chemotherapy followed by IFRT.

CD 20 is a transmembrane protein detected on the surface of most mature B cells. Reed Sternberg cells of classical HD originate from germinal center B cells [19]; however, they seem to have lost their ability to express B cell markers such as CD 20 on their cell surface,leading to a low positivity rate, which commonly ranges from 20–30% [20, 21] although a wide range of positivity of less than 5% to more than 50% has been recorded in some studies [19]. Most authorities, however, would now exclude the diagnosis of classical HL in the presence of more than 20% CD20 homogeneous or strongly positive and will favor in these instances diagnoses of lymphocyte-predominant HL, T-cell/histiocyte-rich large B-cell lymphoma, diffuse large B-cell lymphoma, primary mediastinal large B-cell lymphoma or the gray zone lymphoma [21]. In the index study, only 17% of patients with HL were tested for CD20, and the prevalence of CD20-positive HL was 6.8%; however, it was impossible to ascertain the positivity percentage. The study's poor treatment outcome could be due to misdiagnosis, as only a few patients were tested for CD20.

Overall survival was not affected by either CD20 status or rituximab use.The influence of CD20 status on the treatment outcome is controversial.Benharroch et al. in Israel found no impact of CD20 on overall survival or disease disease-free survival [21] while Khadega et al. in Saudi Arabia similarly established that both CD20 status and rituximab use do not influence HL treatment outcome [22]. Two other studies done earlier [19, 23] were in line with this. Conversely, Memorial Sloan-Kettering Cancer Center found CD20 positive patients had a poorer OS [24] while Tzankov et al. found a better outcome with CD20 positivity in cHL patients. However, Tzankov et al. found that the prognostic significance of CD20 that was observed in the years 1974–1980 disappeared in the subsequent years 1981–1999, suggesting that improved treatment approaches can countervail the importance of CD20 as a prognostic marker [20].

The two-year OS rate of HIV-HL (34%) was almost half that of non-HIV-HL (67%) in the index study. In high-income countries (HIC) like Britain and Germany, recent studies have shown that HIV-HL patients' survival rates are approaching those of non-HIV-HL [9, 10]. However, in the US, Olszewski et al. still found that an unadjusted 5-year overall survival was significantly lower for HIV-HL (66%) than for non-HIV-HL (80%) populations.Among 2090 HIV-HL patients, 81% received chemotherapy, but 16% received no treatment.Advanced age, male gender, non-white race, poor socioeconomic status, and undetermined histologic subtype were associated with a higher risk of non-treatment. However, among patients who received chemotherapy, HIV-positive status was not significantly associated with higher mortality in classical histologic subtypes, including nodular sclerosis and mixed cellularity,leading to the conclusion that worse survival statistics are driven by lower rates of chemotherapy administration among the HIV-HL patients. The prognosis was significantly worse in cases with undetermined histology, suggesting that this subgroup has more aggressive biological or other high-risk characteristics [11]. Relatedly,in this study, it was found that the majority of HIV-HL patients (60.9%) were given fewer than four chemotherapy cycles (de-escalated therapy), which contributed to the observed low OS rate.The poor tolerance of chemotherapy and financial constraints caused HIV-HL patients to receive fewer chemotherapy cycles.Moreover, 22% of patients with HIV-HL were not receiving HAART at diagnosis of HL, indicating poor access to HAART.Only 4.3% of HIV-HL patients received IFRT, while 18.3% of non-HIV-HL received IFRT, suggesting that there is unequal access to radiotherapy according to HIV status.

The HIV-HL patients were found to have higher lactate dehydrogenase (LDH) at presentation compared to non-HIV-HL patients. LDH is a surrogate marker of tumor burden in HL [9, 16, 25]. The higher tumor burden could be another factor that contributes to poorer OS among HIV-HL patients. There was no difference in clinical stages between HIV-HL and non-HIV-HL patients, possibly because of inaccurate staging without using a PET-CT scan.

We found additional predictors of survival in the HIV-HL cohort include CD4 count levels, HAART use, and the duration of HAART use.Both the use of HAART and the duration of use for more than ten months led to significant improvements in survival (*P* = 0.000).In Spain, patients on HAART were found to have significantly higher OS and DFS than non-HAART patients [26]. In France, it was observed that patients diagnosed with HIV-HL after the initiation of HAART lived much longer than those diagnosed before the initiation of HAART [26]. HAART use is associated with improved immunity(CD4 count) and better virologiccontrol, reducing AIDS-associated morbidity and mortality in patients with HL [26]. In the index study, lower CD4 counts were associated with poorer outcomes. Patients with a CD4 count < 200/mm3 had a lower two-year survival rate (*P* = 0.05). A lower CD4 count is linked to a higher chance of developing opportunistic infections [27, 28] and a diminished tolerance to chemotherapy [29]. Bryant et al. in the USA found that low CD4 counts were associated with more hematological toxicities [30].

The retrospective nature and short follow-up period of this study limit its scope.Treatment response and progression-free survival could not be determined due to the lack of a PET-CT scan.A small number of HIV-HL cases were found in the study.It was not possible to assess more survival predictors that are specific to HIV cases, such as viral load and HAART type.Only a few patients were tested for CD 20; toxicology data was not obtained.Although this study is a single-center study, it reveals the importance of linking HIV care with cancer care and the difficulty in accessing care in resource-limited countries.

In conclusion, the prevalence of HIV-HL was 27.7% among HL patients. About 22% of patients were not on HAART upon diagnosis of HIV-HL. HIV positivity is still a poor prognostic factor in our setting, especially for patients not on HAART, on HAART for ≤ 10 months, or with a low CD4 count < 200 cells/mm3. Patients with HIV-HL were older and had higher LDH levels, whereas patients with non-HIV-HL were younger and had low LDH levels.

## Data Availability

The dataset used and analyzed during the current study is available from the corresponding author upon reasonable request.

## Abbreviations

ABVD: Adriamycin, Bleomycin, Vinblastine, Dacarbazine
CHL: Classic Hodgkin’s Lymphoma
IFRT: Involved field radiotherapy
PLWHA: People living with HIV/AIDS
